# Shape of an obstacle affects the mediolateral trajectory of the lower limb during the crossing process

**DOI:** 10.3389/fspor.2023.1130332

**Published:** 2023-08-11

**Authors:** Yuka Miura, Kohei Yoshimoto, Masahiro Shinya

**Affiliations:** ^1^ Graduate School of Humanities and Social Sciences, Hiroshima University, Hiroshima, Japan; ^2^ Research Team for Social Participation and Community Health, Tokyo Metropolitan Institute of Gerontology, Tokyo, Japan

**Keywords:** obstacle avoidance, shape of obstacle, clearance, lower limb, mediolateral trajectory

## Abstract

In previous studies involving obstacle crossing, vertical foot clearance has been used as an indicator of the risk of contact. Under normal circumstances, individuals do not always cross over obstacles with the same height on both sides, and depending on the shape of the obstacle, the risk of contact may differ depending on the foot elevation position. Therefore, we investigated whether task-related control of the mediolateral foot position is adapted to the shape of the obstacle. Sixteen healthy young adults performed a task in which they crossed over two obstacles with different shapes while walking: a trapezoidal obstacle and a rectangular obstacle, as viewed from the frontal plane. It was shown that when crossing over a trapezoidal obstacle, the participants maintained foot clearance by controlling the mediolateral direction, which chose the height that needed to be cleared. The results of this study suggest that the lower limb movements that occur during obstacle crossing are controlled not only in the vertical direction but also in the mediolateral direction by adjusting the foot trajectory to reduce the risk of contact. It was demonstrated that control was not only based on the height of the obstacle directly under the foot but also in the foot mediolateral direction, considering the shape of the entire obstacle, including the opposite limb.

## 1. Introduction

Falls can have a deleterious effect on health, independence, and quality of life across all ages (1). One of the causes of falling is contact with obstacles (2–5). To predict and prevent falls, scientists have investigated the foot trajectory during obstacle crossing (6). In previous obstacle-crossing studies, the vertical foot clearance i.e., the vertical distance between the foot and an obstacle, has been used as a main kinematic outcome (6–9), and clearance has been used to assess the risk of contact between obstacles and the foot. It was shown that clearance was affected by the risk of falling (10), and the effects of aging (8). Clearance during obstacle crossing has also been used as an indicator of improved adaptive walking ability in stroke patients (11). Based on these findings, this parameter is often used in clinical practice as a measure of walking ability associated with certain diseases or aging.

It is known that foot clearance depends on the obstacle's properties such as its height and location (12, 13). However, it has also been shown that clearance is influenced by the environment in which walking is performed. Previous studies have evaluated lower limb motion during the crossing of obstacles based on clearance, which is the vertical distance between the toes and the obstacle. Crossing over obstacles is a movement that is also affected by the pattern of the obstacle, and it is clear that when the pattern of the obstacle is vertical, the clearance is greater than when the pattern is horizontal (14). In addition, the color contrast of obstacles was shown to be another factor that impacted clearance. It has been reported that the high contrast of the colors black and white resulted in greater clearance (15). Thus, depending on the characteristics of an obstacle, humans attempt to reduce the risk of contact by raising their feet vertically to safely cross it. In contrast, Heijnen et al. (16) reported that repeated obstacle-crossing tasks reduced clearance. Moreover, they showed that successfully crossing over an obstacle is based on an energy minimization strategy. Their results suggest that foot clearance is based on a tradeoff between the energy consumption required to control the lower limb and the risk of contact with an obstacle. Heijnen et al. (16) reported on obstacle crossing with minimal clearance in an environment where safety was prioritized, such that even if the feet came into contact with an obstacle during straddling, falling was avoided. In contrast, Shinya et al. (17) reported that in a stair-climbing task, clearance was higher when the risk of contact was higher, in which the memory of stair height was affected by an averted gaze.

**Figure 1:**
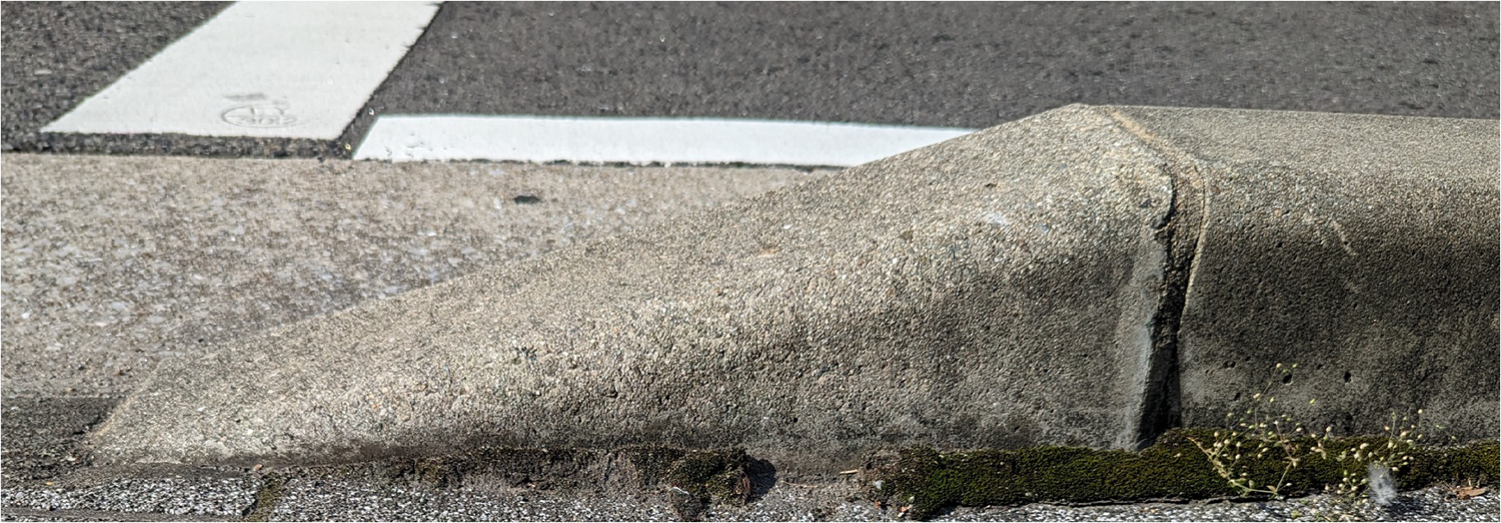
Examples of trapezoidal obstacles encountered under normal circumstances: curbs.

Most previous studies focused on the foot trajectory in the sagittal plane (i.e., vertical foot clearance). However, the influence of crossing an obstacle on the mediolateral foot position during walking should also be investigated. It is necessary to examine the control of the foot trajectory in the mediolateral direction according to the shape of an obstacle, which has not been previously investigated. In the overpass gait, to reduce the risk of falling owing to contact with an obstacle, the foot is elevated vertically. However, Yamagata et al. (2020) reported that excessive foot elevation in the elderly could induce mediolateral instability during obstacle-crossing. This suggests that foot elevation is not always a safe method of crossing a trapezoid. Most people regularly encounter circumstances in which an obstacle must be crossed. However, obstacles are not necessarily the same height on both sides. For example, in the case of a curb (Figure 1) that has an incline and different heights on the left and right sides, the height of this obstacle varies depending on the lateral position of the foot when it is crossed. The crossing of obstacles is an action that is greatly influenced by vision. Miura et al. (18) reported that the clearance of an obstacle is affected by the height that the opposite leg crosses. This suggests that the shape of the obstacle may also affect foot trajectory. Therefore, it is possible that clearance could be facilitated by moving the foot mediolaterally, rather than raising it vertically, depending on the shape of the obstacle. In previous studies involving obstacle-crossing, experiments were conducted using rectangular obstacles of uniform height on both sides. When crossing a rectangular obstacle, the risk of contact can be reduced by controlling the height of the foot in the vertical direction without moving it mediolaterally. However, when crossing an irregularly shaped obstacle such as a trapezoid, the foot must be controlled in the vertical direction, which could induce instability of the body in the lateral direction. We assert that it is necessary to determine whether foot control in the mediolateral direction, as well as the vertical direction, is necessary during obstacle crossing, depending on the shape of the obstacle.

Lower limb movements during obstacle crossing are based on energy minimization strategies (16), and Slawinski et al. (19) reported that in healthy participants, higher foot elevation corresponded to greater energy expenditure. Although there are no published reports on the quantitative estimation of the energy expenditure associated with the MP1 vertical position during obstacle crossing, previously published results by Slawinski et al. (2020) suggested that energy expenditure increases with foot elevation in healthy individuals. This implies that the MP1 vertical position might achieve the same foot clearance more efficiently in the mediolateral direction than in the vertical direction. Therefore, the risk of contact with an obstacle and energy consumption must be considered in obstacle-crossing, in addition to postural stability. Yamagata et al. (20) reported that excessive foot elevation in obstacle-crossing could induce postural instability in the mediolateral direction. Accordingly, a safer strategy for the crossing of irregularly shaped obstacles should be adopted, such as increasing the MP1 position in the mediolateral direction to reduce the risk of contact with the obstacle, instead of elevating the foot to achieve the same outcome.

**Figure 2:**
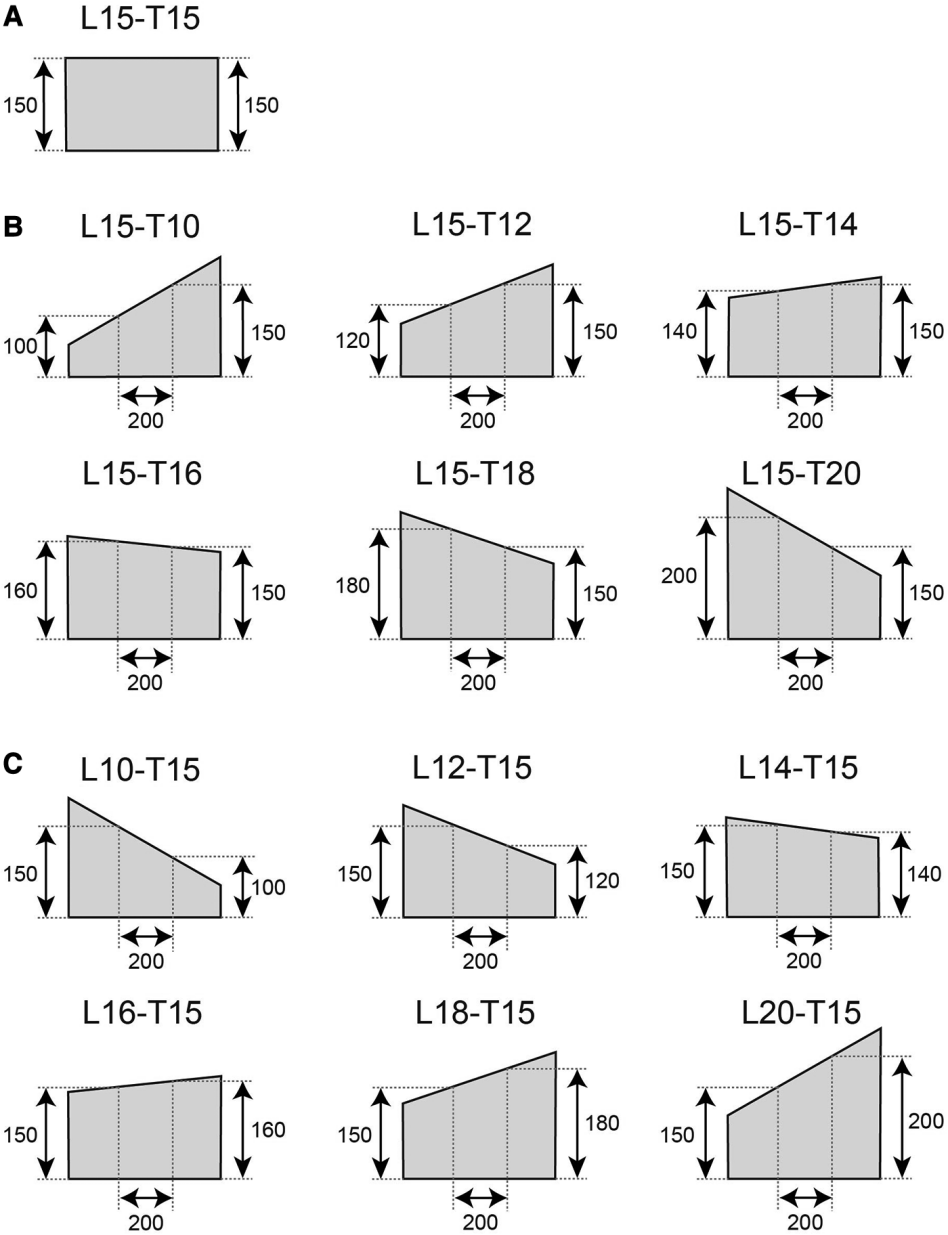
Geometry of the obstacles. A rectangular 150-mm-height obstacle with a flat top edge was used as a reference (**A**). Trapezoidal obstacles in which the height of the leading limb was fixed at 150 mm (**B**). Height of the trailing limb was set from 100 mm to 200 mm. (**C**) Trapezoidal obstacles in which the height of the trailing limb was fixed at 150 mm (**C**).

In previous studies, since the obstacle height was generally the same in the mediolateral direction, foot clearance and the risk of collision did not change with the mediolateral foot movement. Thus, the control of foot position was relevant only in the vertical direction. In contrast, if an obstacle is trapezoidal in the frontal plane and the top edge is tilted in the mediolateral direction, the mediolateral foot position would be task-relevant. For example, if the top edge of an obstacle is in the right-up-left-down position, moving the foot leftward would increase the clearance even if the vertical elevation is the same. As such, it is not clear if the mediolateral foot position is influenced during obstacle-crossing. Therefore, using rectangular and trapezoidal obstacles, we aimed to examine whether task-related control of mediolateral foot position adapts to the shape of an obstacle by comparing the trajectory of the foot when crossing rectangular and trapezoidal obstacles (Figure 2). Slawinski et al. (18) reported that foot elevation increases energy expenditure in healthy adults. Although there are no published reports that directly compared the energy consumption of mediolateral foot movement with foot elevation, it can be assumed that moving the foot laterally requires less energy than elevating it, at least in healthy young adults. In addition, the finding that clearance decreases with the number of trials in over-the-obstacle gait suggests that lower limb movement in this case is based on an energy minimization strategy (16). Therefore, based on these findings, we hypothesized that individuals control their feet not only vertically but also laterally, based on the shape of the obstacle.

## 2. Methods

### 2.1. Participants

The inclusion criteria for participants of the study were the absence of impediments to normal locomotion and normal or corrected-to-normal vision. Neurological or musculoskeletal disorders were the main exclusion criteria. An *a priori* power analysis was performed based on repeated ANOVA tests using G*power 3.1.9.7. The parameters for the power analysis were set as follows: effect size f = 0.30, alpha = 0.05, 1-beta = 0.8, number of groups = 1, number of measurements = 7, correlation among measures = 0.50, and non-sphericity correction e = 1. The effect size f was obtained from the vertical clearance observed in our previous report (18). The suggested sample size was 12. Conservatively, we recruited sixteen healthy young volunteers (eight males/eight females; age: 21.3 ± 1.7 years; height: 165.6 cm ± 7.9 cm; weight: 59.6 ± 9.9 kg, mean ± standard deviation). All subjects gave written informed consent before participation, and the ethics committee of the Graduate School of Integrated Arts and Sciences, Hiroshima University, approved the study (approval number: 02–30) according to the Declaration of Helsinki.

### 2.2. Experimental protocol

Each volunteer walked at a self-selected pace on a 7 m walkway and crossed an obstacle. The volunteers were instructed to walk upright and cross the obstacle with the right (leading) limb during the 7th step and the left (trailing) limb during the 8th step. An obstacle was placed in the middle of the walkway. The obstacles were placed in the center of the laboratory. To avoid the feet of the participants from passing through the center of the obstacle, a black tape was placed in the middle of the walking path. The participants were instructed to not step on or step over the tape. Before the recording session, the volunteers were instructed to adjust the starting position without crossing the obstacle. We used rectangular and trapezoidal obstacles as illustrated in Figure 2. The obstacle was made of Styrofoam and was 1.5 cm in depth. The height of the rectangular obstacle was 15 cm. As both the leading and trailing limbs were expected to cross a height of 15 cm, we referred to the rectangular obstacle as L15-T15. In one group of the trapezoidal obstacles, the height at 10 cm to the right (+10 cm in the x-coordinate) from the middle of the obstacle was fixed to 15 cm, and the height at 10 cm to the left (−10 cm in the x-coordinate) from the middle of the obstacle was set to 10 cm, 12 cm, 14 cm, 16 cm, 18 cm, and 20 cm (Figure 2B). We referred to these trapezoidal obstacles as L15-T10, L15-T12, L15-T14, L15-T16, L15-T18, and L15-T20, respectively. Similarly, we created another group of trapezoidal obstacles (L10-T15, L12-T15, L14-T15, L16-T15, L18-T15, and L20-T15) of which the height at 10 cm to the left of the center was fixed at 15 cm, and the height at 10 cm to the right of the center was set to 10 cm, 12 cm, 14 cm, 16 cm, 18 cm, and 20 cm (Figure 2C). Note that the obstacle heights used in this study are the height values at 10 cm from the center of the obstacle on either side. The vertices of the trapezoidal obstacles were set higher than the height of the obstacles used in this study at 10 cm from the center to the left and right. The experiment was block-designed and the participants repeated 10 trials for a given obstacle session before moving to another obstacle session. The order of the sessions varied among the participants. For each shape, 10 trials were performed, and each participant completed a total of 150 trials across the 15 conditions, with 10 trials per condition. Sessions 1, 8, and 15 used rectangular obstacle (15–15) sessions. For eight participants, Sessions 2–7 used group B trapezoidal obstacles (15–**), and Sessions 9–14 used group C obstacles (**−15). For the other eight participants, the order of the group B and C trapezoidal obstacles was the opposite. In Sessions 2–7 and 9–14, the order of the obstacles was randomized such that the right-side-down and left-side-down obstacles were alternated (the experimental order is listed in the Supplementary Table S5).

### 2.3. Data collection

Reflective markers were pasted on four anatomical landmarks on the left and right distal condyles of the first (MP1) and fifth (MP5) metatarsal bone, respectively. The markers were captured using a 3D optical motion caption system (Qualisys Track Manager, Qualisys, Göteborg, Sweden) with eight cameras (Qualisys-Miqus M3, Qualisys). The sampling frequency of the kinematic data was 250 Hz and the measured signals were stored on a computer. All numerical calculations were performed using MATLAB 2017b (Math Works, Inc., MA, USA).

### 2.4. Data analysis

**Figure 3:**
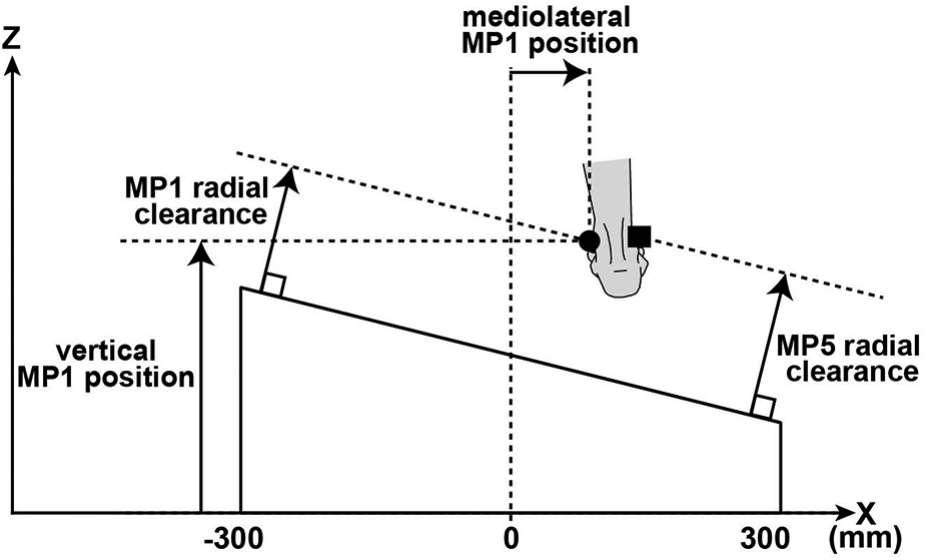
Illustration of radial foot clearances in the frontal plane. The foot clearance was calculated based on the MP1 (black circle) and the MP5 (black square) markers.

In this study, the participants did not practice crossing over the obstacles before the experiments. Therefore, the first 10 trials in which participants crossed a rectangular obstacle were excluded from the analysis. In addition, the left leg was the leading limb in 2 trials. The left toe marker could not be measured because it could not be captured by the camera used for this trial. The participants came into contact with the obstacles in 10 trials. These trials were excluded from the analysis. The kinematic data were low-pass filtered using a zero-lag second-order Butterworth digital filter with a cutoff frequency of 10 Hz. The foots trajectory during obstacle crossing was quantified for the leading and trailing limbs using the following parameters (Figure 3). The vertical MP1 position was defined as being measured from the floor. The mediolateral MP1 position was in the mediolateral direction with the center of the obstacle as the origin. The MP1 radial clearance was the shortest distance from the obstacle to the MP1 marker and the MP5 radial clearance was the shortest distance from the obstacle to the MP5 marker.

### 2.5. Statistics

One-way repeated measures ANOVA was used for all dependent variables to examine the effect of the obstacle's shape on the foot trajectory of the lower limb. Bonferroni correction was used for *post hoc* comparisons; if it was determined that there was a violation of sphericity using Mauchly's test, a Greenhouse-Geisser correction was performed. Significance was set *a priori* to p < 0.05. A total of six *post hoc* comparisons were conducted to compare rectangular obstacles to trapezoidal obstacles. The significance level for *post hoc* comparisons was set at p < 0.0083 (0.05/6). Cohen's d values of 0.2, 0.5, and 0.8 were regarded as small, medium, and large effects, respectively (21). *η*^2^ values of 0.01, 0.06, and 0.14 were considered to be small, medium, and large effects, respectively (21). Statistical analysis was performed using JASP version 0.16.1.0 (Eric-Jan Wagenmakers, Amsterdam, Netherlands).

## 3. Results

In this study, the lower limb movements during the process of crossing an obstacle were controlled not only in the vertical direction but also in the mediolateral direction by controlling the foot trajectory to reduce the risk of contact. The results for the vertical MP1 position, mediolateral MP1 position, and MP1 and MP5 radial clearance are presented in the following section.

**Figure 4:**
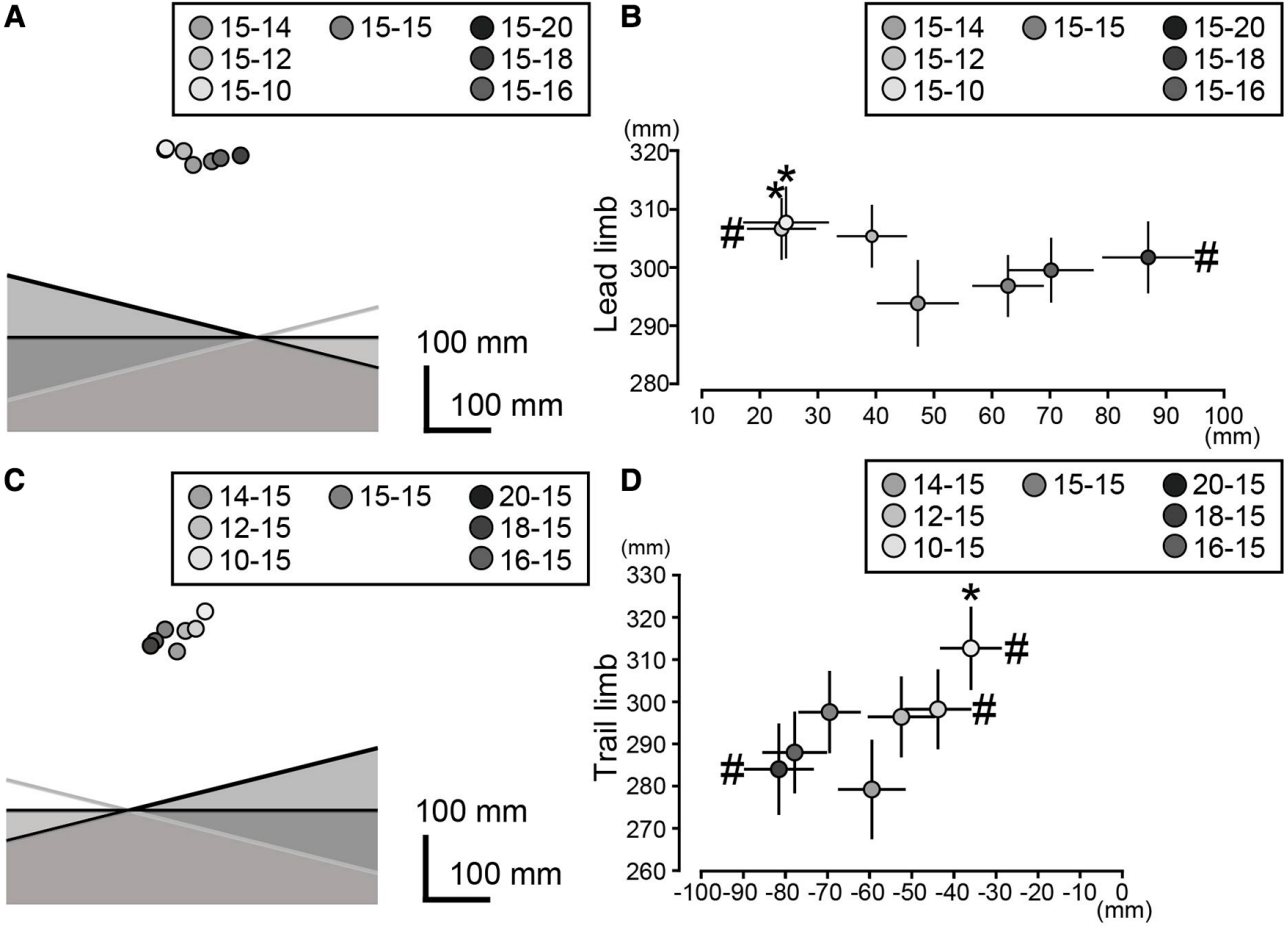
Vertical and mediolateral MP1 positions of the leading limb (**A,B**) and trailing limb (**C,D**). In the left panels, the mean MP1 positions for all the obstacle conditions are shown. To visualize the foot position relative to the obstacle, three obstacles (15-10, 15-15, and 15-20 in panel A; 10-15, 15-15, and 20-15 in panel C) are illustrated. The right panels show the mean and standard deviations of the foot position (**B,D)**. The asterisks and hash marks indicate significant differences for the rectangular 15-15 obstacles in the vertical and mediolateral MP1 positions, respectively. The significance level was adjusted to *p* < 0.0083 (0.05/6) based on the Bonferroni correction.

Repeated measures ANOVA tests revealed significant influential effects of the obstacle conditions on the vertical MP1 position in the leading and trailing limbs. (leading limb: *F_(2.804,42.065)_ = 3.823, p = 0.018, η^2^ = 0.203*; trailing limb: *F_(6, 90)_ = 6.363, p < .001, η^2^ = 0.298*). Based on the *post hoc* test, the vertical MP1 position of the leading limb in the L15-T10 and L15-T12 was larger compared to that of the rectangular obstacle (i.e., L15-T15). (L15-T10:*t(15) = 3.669, p < 0.0083, Cohen's d = 0.374;* L15-T12: *t(15) = 3.38, p < 0.0083, Cohen's d = 0.345*) (Figure 4B). The vertical MP1 position of the trailing limb in the L10-T15 was larger than that of the rectangular obstacle [L10-T15: *t(15) = 5.377, p < 0.0083, Cohen's d = 0.672*] (Figure 4D).

Significant influential effects of the obstacle conditions on the mediolateral MP1 position were observed for the leading and trailing limbs (leading limb: *F_(2.436, 36.535) _= 18.244, p < .001, η^2^ = 0.549*; trailing limb: *F_(1.866, 27.997) _= 20.301, p < .001, η^2^ = 0.575*). There were significant differences between L15-T12 and L15-T15, and between L15-T20 and L15-T15 in the leading limb (L15-T12: *t(15) = −3.341, p < 0.0083, Cohen's d = −0.591*; L15-T20: *t(15) = −4.814, p < 0.0083, Cohen's d = −0.852*) (Figure 4B). There were also significant differences between L10-T15 and L15-T15, L12-T15 and L15-T15, and L20-T15 and L15-T15 in the trailing limb (L10-T15: *t(15) = 4.554, p < 0.0083, Cohen's d = 0.683*; L12-T15: *t(15) = 3.767, p < 0.0083, Cohen's d = 0.565*; L20-T15: *t(15) = 3.467, p < 0.0083, Cohen's d = 0.52*) (Figure 4D). These results indicate that both the leading and trailing limbs were consistently displaced in the direction of the low height of the obstacle during the crossing of the trapezoidal obstacles.

**Figure 5:**
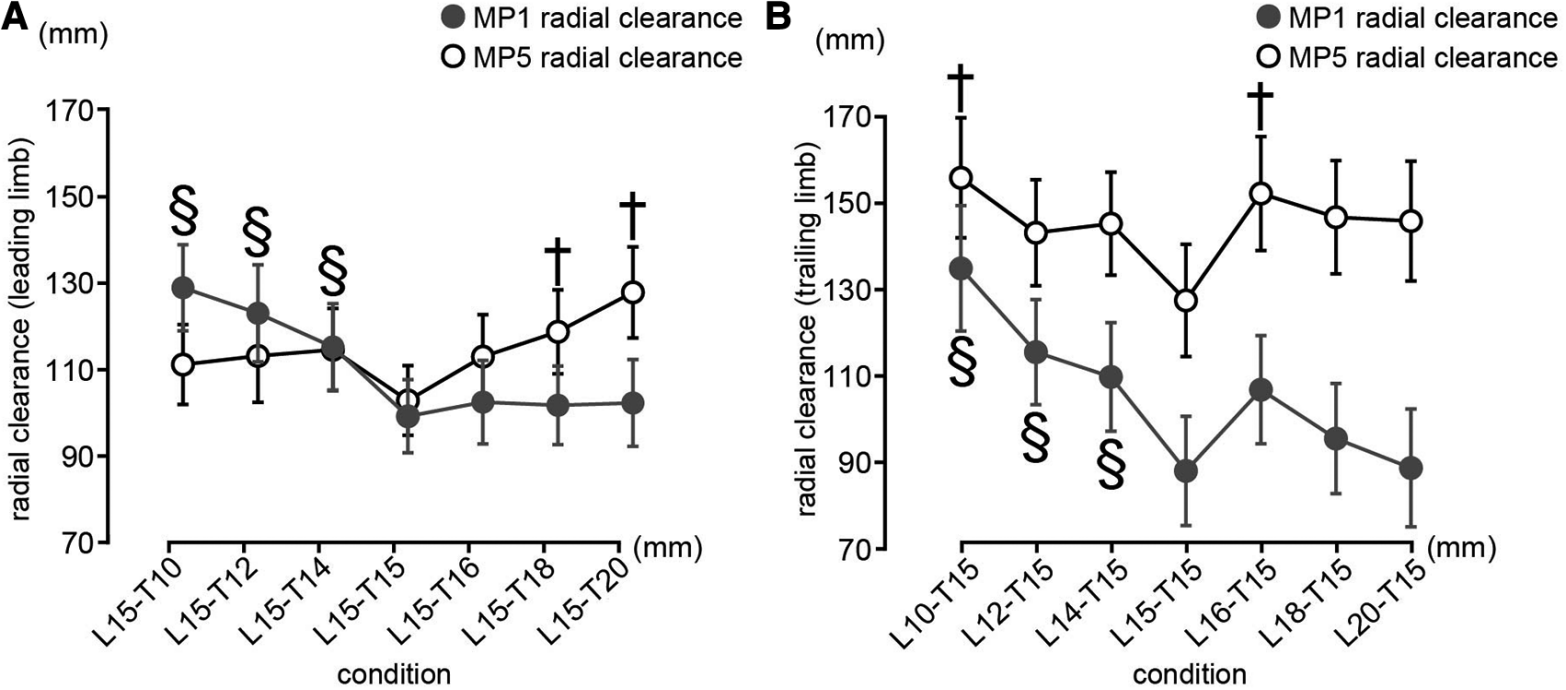
Mp1 and MP5 radial clearances of the leading limb (**A**) and trailing limb (**B**). (**A**) The vertical axis shows the radial clearance of the leading leg and the horizontal axis shows the height of the obstacle on the trailing leg side. (**B**) The vertical axis shows the radial clearance of the trailing leg and the horizontal axis shows the height of the obstacle on the (**A**) The vertical axis shows the radial clearance of the leading leg and the horizontal axis shows the height of the obstacle on the trailing leg side. The mean and the 95% confidence intervals are illustrated. The daggers indicate significant differences for the rectangular obstacle (150 mm condition) for MP1 radial clearance with *p* < 0.0083 (0.05/6). The section describes the significant differences of the rectangular obstacle (150 mm condition) in the case of MP5 radial clearance with *p* < 0.0083 (0.05/6).

Significant influential effects related to the obstacle conditions on the MP1 radial clearance were observed in the leading and trailing limbs (leading limb: *F_(6, 90)_ = 21.948, p < .001, η^2^ = 0.594*; trailing limb: *F_(6, 90)_ = 15.607, p < .001, η^2^ = 0.510*). A significantly larger MP1 radial clearance was observed in the L15-T10, L15-T12, and L15-T14 compared to that of the rectangular obstacle in the leading limb (L15-T10: *t(15) = 8.248, p < 0.0083, Cohen's d = 0.808*; L15-T12: *t(15) = 6.61, p < 0.0083, Cohen's d = 0.648*; L15-T14: *t(15) = 4.45, p < 0.0083, Cohen's d = 0.436*) (Figure 5A), and L10-T15, L12-T15, and L14-T15 compared to that of the rectangular obstacle in the trailing limb (L10-T15: *t(15) = 7.843, p < 0.0083, Cohen's d = 0.961*; L12-T15: *t(15) = 4.6, p < 0.0083, Cohen's d = 0.564;* L14-T15: *t(15) = 3.64, p < 0.0083, Cohen's d = 0.446*) (Figure 5B).

There was a significant influential effect of the obstacle conditions on the MP5 radial clearance in the leading and trailing limbs (leading limb: *F_(6, 90)_ = 7.259, p < .001, η^2^ = 0.326*; trailing limb: *F_(6, 90)_ = 4.071, p = 0.001, η^2^ = 0.213*). A significantly larger MP5 radial clearance was observed in the L15-T18 and L15-T20 compared to that of the rectangular obstacle in the leading limb (L15-T18: *t(15) = −3.977, p < 0.0083, Cohen's d = −0.437*; L15-T20: *t(15) = −6.261, p < 0.0083, Cohen's d = −0.688*) (Figure 5A). In addition, a significantly larger MP5 radial clearance was observed in the L10-T15 and L16-T15 compared to that of the rectangular obstacle in the trailing limb (L10-T15: *t(15) = 4.508, p < 0.0083, Cohen's d = 0.58*; L16-T15: *t(15) = −3.931, p < 0.0083, Cohen's d = −0.505*) (Figure 5B).

## 4. Discussion

We investigated whether task-related control of the mediolateral foot position is adapted to the shape of the obstacle. The hypothesis was confirmed based on the results that when the obstacle was left-up-right-down, the foot passed rightward compared to the case of the flat obstacle and vice versa for the right-up-left-down obstacles. The foot trajectory during obstacle crossing was determined by considering the tradeoff between energy minimization and the reduction of the risk of contact with the obstacle (16). Physiological energy increases in proportion to the lifting height during obstacle-crossing (19). Although there are no published reports that quantitatively estimate the energy expenditure associated with the mediolateral MP1 position during obstacle crossing, it is possible that at least for healthy young participants, the mediolateral MP1 position is more efficient than vertically lifting the foot to ensure foot clearance. The observed mediolateral movement of the foot might be regarded as a strategy for achieving clearance when crossing trapezoidal obstacles. In addition, the action of crossing obstacles relies heavily on vision. Clearance is affected by the pattern and color contrast of the obstacle (14, 15). In addition, lower limb movements are regulated by the overall shape of the obstacle, including the opposite leg (18). In this study, the height of the vertex of the obstacle was higher than the height at 10 cm from the center of the obstacle on either side. It was considered that the movement of the foot in the mediolateral direction was influenced not only by the height of the obstacle directly below the foot but also by the height of the vertex of the obstacle.

A significantly larger MP1 radial clearance was observed in the case of the leading limb when the participants crossed the L15-T10, L15-T12, and L15-T14 trapezoidal obstacles compared to the 15-cm-high rectangular obstacle. For these obstacles, the lateral side was higher than the medial side, resulting in an increased risk of contact of the lateral side of the foot with the obstacles. In these cases, the radial clearance that was determined using the MP5 marker was not significantly different from that obtained for the rectangular obstacle condition. Conversely, the MP1 radial clearance for the L15-T16, L15-T18, and L15-T20 obstacles was not significantly different from that of the rectangular obstacle, whereas the MP5 radial clearance for the L15-T18 and L15-T20 obstacles was larger compared to that of the rectangular obstacle. Overall, the smaller value of the radial clearance of MP1 and MP5 for the trapezoidal obstacles was not statistically different compared to the foot clearance in the case of the rectangular obstacles. A similar result was confirmed for the trailing limb. These results suggest that the participants achieved foot clearance by considering not only the height of the obstacle that is crossed when the foot is at its nadir but also the height of the obstacle that the opposite limb crosses. Thus, the risk of impact of both the inside and outside of the foot with the obstacle is constant.

As previously indicated, the foot clearance for the trapezoidal obstacles was not significantly different from that for the rectangular obstacle. The only exception was the L10-T15 condition, in which both the MP1 and MP5 radial clearances for the trailing limb were larger compared to that of the reference foot clearance for the rectangular obstacle. Crossing obstacles while walking is dependent on the visual information stored in the brain (22). The position of the trailing limb relative to an obstacle cannot be confirmed using online visual information. This means that the control of the trailing limb is more uncertain compared to the leading limb. Previous studies have reported an increase in foot clearance in the case of high uncertainty, such as when the subject's gaze was averted from a stair for 2 s or more (17). In the L10-T15 condition, the obstacle's height was higher on the lateral side of the trailing limb, wherein the risk of collision could be higher for the lateral side of the foot. A previous psychological study demonstrated that humans tend to misjudge their foot position as being more medial compared to their actual foot position and the authors suggested that this discrepancy might lead to unexpected tripping during walking (23). If the perception of the location of the lateral side of the foot is uncertain, the results indicate that the participants increase foot clearance when the risk of tripping was considered as part of a strategy for adaptive locomotion. It is important to note that the motion of the leading limb is not equal to that of the trailing leg. Moreover, it has been reported that when crossing an obstacle taking an opaque box, only the clearance of the leading limb increased compared to the unloaded and transparent box condition, owing to the narrowing of the visual field (24). These findings suggest that the observed behavior differs between the leading and trailing limbs. However, it was also reported that during steeping over an obstacle in a VR environment, the leading and trailing limbs share motor memories based on visual input (25) and that the motion of the trailing limb is determined based on the memory of the obstacle height before the leading limb crossed the obstacle (26). Therefore, it should be noted that the finding that the motion of the leading and trailing limbs is not equal, requires further examination in the field of obstacle-crossing walking, given that there are different claims. McFadyen et al. (27) found that vestibular system stimulation during obstacle crossing caused lateral changes in body orientation, but this did not affect sagittal foot trajectory including clearance. The absence of a significant difference in radial clearance between rectangular and trapezoidal obstacles in this study suggests that sensory information, such as vestibular and visual cues, may influence lower limb movements during obstacle crossing. However, the impact of sensory information could not be determined in this study because it was not quantified.

Although the results of this study are qualitatively reasonable, it might be premature to conclude that they are optimal from a quantitative perspective. The basic principle of motor control is energy minimization (16). If this principle is applied to the experimental results, the observed mediolateral MP1 position can be regarded as an energy-efficient strategy to ensure foot clearance compared to the vertical MP1 position, which guarantees the same clearance. In contrast, it was reported that even in an environment where physical safety was maintained, such as crossing a fragile obstacle, greater clearance was observed compared to crossing a less fragile obstacle (28). Nevertheless, it should be noted that the energy minimization strategies discussed in this study are theoretical and still under debate in the scientific community. It would be interesting to investigate the foot trajectory in the frontal plane of elderly individuals, amputees, and patients diagnosed with stroke or Parkinson's disease. For these populations, it can be assumed that the motor cost of lifting the foot is higher, the balance-maintaining ability is reduced, the result of tripping is more serious, and perception and memory-related functions are impaired compared to healthy people (7, 12, 29). Crossing a trapezoidal obstacle is a complex task that requires the integration of many functions. Ambulatory obstacle-crossing has been reported to be different in patients with Parkinson's disease compared to healthy individuals, with increased asymmetry in the left and right legs (7) and greater clearance in stroke patients (30). These findings suggest that trapezoidal obstacle crossing in other populations (e.g., osteoarthritis patients, hemiplegic patients, and individuals with other musculoskeletal and neurological disorders) may differ compared to healthy individuals. Although healthy individuals can control their lower limbs in the mediolateral direction considering the overall shape of the obstacle, even when crossing a trapezoidal obstacle, patients with certain diseases may engage in different motor strategies compared to healthy individuals when crossing these obstacles. In future research, obstacle crossing should be investigated in other populations with different locomotor costs to determine how the disease may affect obstacle crossing in complex environments. It is important to note that one of the limitations of this study is that it is unclear whether the change in the mediolateral position of the foot during the crossing process was primarily controlled by the trunk or by hip abduction and internal rotation, or if it resulted from leaning during the stance phase. Further studies are required to analyze the underlying mechanism of the mediolateral behavior of the foot when crossing trapezoidal obstacles, including the behavior of the torso and hip joints.

One common cause of falls is tripping over obstacles (3). In daily life, individuals often encounter situations where they must cross obstacles that may not have a uniform height or symmetrical shape. It has been reported that when walking over obstacles, lower limb movements are comprehensively controlled, including the height and the movement of the obstacle, as well as the opposite leg (18). This finding suggests that the overall shape of the obstacle may be a crucial factor in lower limb locomotion during obstacle-straddling gait. When crossing a rectangular obstacle, it is necessary to control the foot trajectory only in the vertical direction. However, when crossing an irregularly shaped obstacle, it is necessary to focus on the control of the foot trajectory not only in the vertical direction but also in the lateral direction. Therefore, it can be inferred that straddling an irregularly shaped obstacle involves more complex control than straddling a uniform obstacle. Individuals perform locomotion not only in a monotonous environment but also in an environment where factors other than shape have complex effects. We propose that by clarifying motor control in complex environments, such as crossing irregularly-shaped obstacles, it is possible to minimize the risk of falls.

## 5. Conclusions

This study confirmed that for a right-up-left-down obstacle, the leading limb passes through the left side of the obstacle toward the foot. Conversely, for a left-up-right-down obstacle, the foot passes through the right side. The same behavior was observed for the trailing limb. These results suggest that the mediolateral position of the feet during obstacle crossing is influenced by the shape of the obstacle. In addition, for the leading limb, the MP5 radial clearance and the clearance obtained for a rectangular obstacle were the same in the case of a right-up-left-down obstacle. Conversely, for a left-up-right-down obstacle, the MP1 radial clearance and the clearance observed for the rectangular obstacle were the same. The same behavior is generally observed for the trailing limb. These results suggest that the participants maintained foot clearance and both the medial and lateral sides of the foot were at risk of collision with the obstacle.

## Data Availability

The raw data supporting the conclusions of this article will be made available by the authors, without undue reservation.
